# The Exploding Aliveness of the World

**DOI:** 10.3201/eid2304.AC2304

**Published:** 2017-04

**Authors:** Byron Breedlove, Paul M. Arguin

**Affiliations:** Centers for Disease Control and Prevention, Atlanta, Georgia, USA

**Keywords:** art science connection, emerging infectious diseases, art and medicine, about the cover, American Modernism, Joseph Stella, public health, infectious diseases, microbes, viruses, bacteria, The Exploding Aliveness of the World, Spring (The Precession) Mesa

**Figure:**
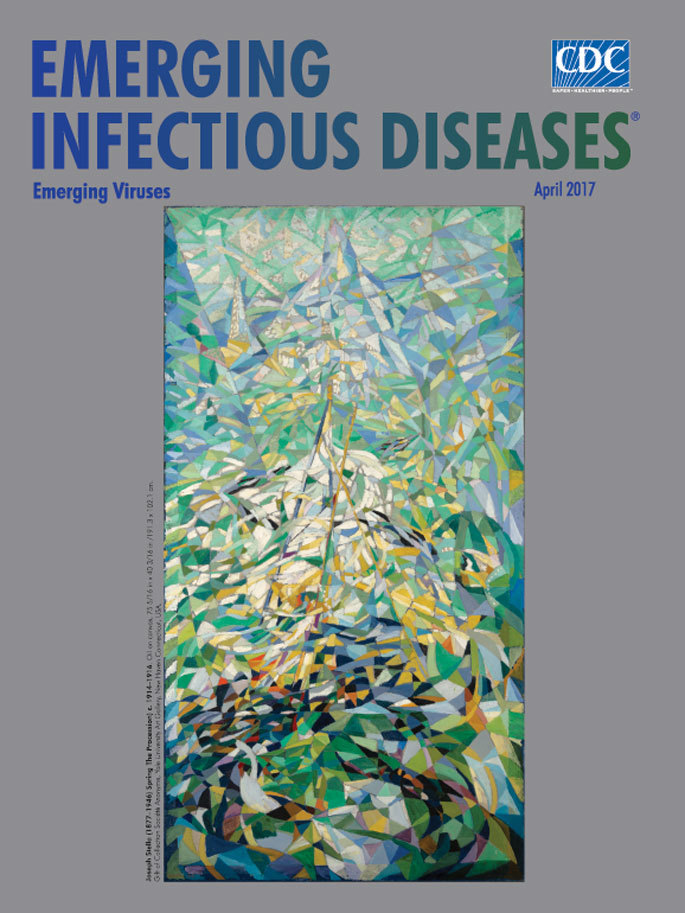
**Joseph Stella (1877–1946), Spring (The Procession) (c. 1914–1916). Oil on canvas, 75 5/16 in × 40 3/16 in/191.3 cm × 102.1 cm. **Gift of Collection Société Anonyme, Yale University Art Gallery, New Haven, Connecticut, USA

When Joseph Stella journeyed to New York City from his native Italy in 1896, he intended to study medicine. Stella instead pursued art, which he studied at the Art Students League of New York under William Merritt Chase. During 1905–1909, Stella illustrated magazines, and he continued to draw throughout a career marked by numerous shifts in his style and approach to art. According to a brief bio from the Phillips Collection website, Stella “began as an academic realist, but his mature work is in a modernist style, notable for its sweeping and dynamic lines.”

During a return visit to Italy in 1909, Stella had his initial brush with modernism, specifically with the Italian Futurist school of painting. According to art critic Holland Carter, “Color entered his art with a bang when he was introduced to Italian Futurism.” Not long after returning to New York from Europe, Stella became immersed in modernism, and he is now viewed as a principal figure in American Modernism.

“Spring (The Procession)” this month’s cover image, a painting bursting with innumerable dazzling slivers of color, is considered among Stella’s finest works. The artist concentrates dark blue, gold, green, and red shards in the lower quarter, suggesting the interactions of biological and chemical processes following the spring thaw. Swirls of pale yellow accented with bursts of green, blue, and gold near the painting’s center reflect the showiness of an early spring garden as the first flowers, shoots, and leaves appear, hungry insects and birds take wing, and lizards and amphibians awaken from their winter torpor. Green, the most common color in nature, dominants Stella’s celebration of renewal. According to art critic Richard Nilsen, “He was one of those painters, like Van Gogh, who yearned to express the exploding aliveness of the world, a man with a visionary sense of cosmic energy.”

“The exploding aliveness of the world” that fuels artistic creativity also finds full expression in dynamic microscopic realms teeming with unfathomable numbers of viruses, bacteria, fungi, prions, and protozoa that lead to an incredible variety of pathologic consequences when infecting their hosts. An editorial in *Nature Reviews Microbiology*, which attempts to quantify some of those numbers used to give perspective to this microscopic world, provides this frame of reference for viruses: “Astronomy is a field that is used to dealing with large numbers, but these can be dwarfed when compared with life on the microbial scale. For instance, if all the 1 × 10^31^ viruses on earth were laid end to end, they would stretch for 100 million light years.” And that is just the viruses!

That only some 1,400 recognized microbes are known to be pathogenic to humans should not be considered reassuring. Since the 1980s, nearly 40 new pathogens have been identified as causes of disease in humans. HIV, Ebola virus, MERS coronavirus, SARS coronavirus, West Nile virus, and Zika virus are among the high-profile viruses that have emerged from the confluence of ecologic forces. Potential new exposure to these previously quiescent microbes can result from human incursion and settlement in remote and rural areas, food production and importation practices, and prevailing planetary weather and temperature.

Comparable to the exuberance of spring, the emergence of microbes is often a spectacular display of the power of nature. Similar to the explosion of springtime pollen, some emerging infections declare their presence with large outbreaks that are impossible to miss. Others are insidiously tenacious, going unnoticed until entrenched like flowing waves of kudzu. Even familiar foes such as *Staphylococcus aureus,* Enterobacteriaceae, and mycobacteria emerge in their own surprising new slivers of color with the development of multiple and extensively drug-resistant varieties.

It has been 25 years since the publication of *Emerging Infections: Microbial Threats to Health in the United States *and 14 years since the publication of *Microbial Threats to Health: Emergence, Detection, and Response*. Those influential reports—which represent the insights of Joshua Lederberg, Robert Shope, and their colleagues—from the Institute of Medicine (now National Academy of Medicine) Committee on Emerging Microbial Threats offered far-reaching recommendations and galvanized support for research and public health action to address the challenges posed by new, emerging, and reemerging infectious diseases. Our success, now and in the future, depends on having a fully functioning national and global public health surveillance system, supported with epidemiologic and laboratory capacity, being able to rapidly share and communicate information.

Springtime is when we see the results of the seeds we have sown. The time and attention given to tending to our backyard gardens, our larger communities, our public health infrastructure, and our approach to addressing emerging infections will be apparent and on display when that inevitable exploding aliveness occurs. Stella’s intoxicating depiction of spring may serve to remind us that we must not become complacent, but that we must constantly renew our focus, thinking, and approaches to addressing emerging infections.
